# A putative prognostic model for lung adenocarcinoma based on crotonylation-related genes by bioinformatics and experimental verification

**DOI:** 10.3389/fcell.2026.1639773

**Published:** 2026-02-17

**Authors:** Wenting Wang, Baixiang Cui, Haoyue Li, Zihan Zhen, Xueqing Song, Xiaoyu Yuan, Qing Cui, Qi Yuan, Yong Liu

**Affiliations:** 1 School of Basic Medical Sciences, Mudanjiang Medical University, Mudanjiang, China; 2 Pathology Department, The Second Affiliated Hospital of Mudanjiang Medical University, Mudanjiang, China; 3 Department of Scientific Research, Mudanjiang Medical University, Mudanjiang, China; 4 Jiamusi University, Jiamusi, China; 5 College of Life Sciences, Mudanjiang Medical University, Mudanjiang, China

**Keywords:** crotonylation, FAM83A, glycolysis, lung adenocarcinoma, prognostic model

## Abstract

**Backgrounds:**

Protein crotonylation is a novel post-translational modification implicated in tumorigenesis and progression. Its putative roles and mechanisms in lung adenocarcinoma (LUAD), however, remain incompletely elucidated.

**Methods:**

We analyzed the expression of crotonylation-related genes (CRGs) in LUAD samples and identified differentially expressed genes for gene set variation analysis (GSVA). Using GSVA scores as phenotypic traits, weighted gene co-expression network analysis (WGCNA) was applied to identify key module genes. A putative prognostic model was subsequently constructed via Lasso-Cox regression. Functional enrichment, gene mutation analysis, and immune infiltration analyses were conducted to compare high- and low-risk groups. Furthermore, cellular experiments were performed to validate the putative role of the hub gene FAM83A and its regulation of key glycolytic enzymes PKM2 and LDHA.

**Results:**

We established a putative crotonylation-related prognostic model for LUAD, which effectively stratified patients into high- and low-risk groups with significantly different overall survival. Functional analysis suggested putative disparities in metabolic pathways between the two groups. Mutation landscape analysis revealed distinct genomic variation patterns, while immune infiltration assessment indicated a putative immune-evasion phenotype in high-risk patients. Cellular assays demonstrated that FAM83A enhances lung cancer cell proliferation, putatively through promoting glycolysis. Our findings establish that FAM83A integrates histone H3K27 crotonylation signaling to drive transcriptional reprogramming of glycolytic metabolism, specifically upregulating key enzymes PKM2 and LDHA.

**Conclusion:**

This study proposes a putative prognostic model based on CRGs for LUAD outcome prediction. The hub gene FAM83A may facilitate lung cancer cell growth by regulating glycolysis via PKM2 and LDHA, offering a novel theoretical foundation and a potential target for prognostic assessment and targeted therapy in LUAD patient.

## Introduction

Lung cancer is a malignant tumor with high morbidity and mortality worldwide, which seriously threatens human health (Su et al., 2025; Thai et al., 2021; Hendriks et al., 2024). Lung adenocarcinoma (LUAD) is one of the most common subtypes of lung cancer, accounting for approximately 40% of lung cancer cases (Denisenko et al., 2018). The complex molecular mechanism and heterogeneity of LUAD have severely restricted the development of precision diagnosis and treatment (Seguin et al., 2022; Xin et al., 2025). The combination of high-throughput sequencing technology and bioinformatics analysis can reveal the molecular characteristics of LUAD, and provide important clues for exploring potential therapeutic targets (Ren et al., 2023; Tang et al., 2024; Wang B. et al., 2025). Therefore, finding effective prognostic biomarkers and therapeutic targets based on transcriptome analysis is of great significance to improve the prognosis of LUAD patients.

Crotonylation modification is a novel post-translational modification of proteins. Similar to the traditional acetylation modification, crotonylation modification is also catalyzed by the corresponding acyltransferases and deacylases, and it has the characteristic of dynamic reversibility (Wu et al., 2018). Recent studies have shown that crotonylation modification is widely present in various biological processes, such as gene transcriptional regulation, cell cycle regulation, metabolic regulation, etc., and plays an important role in maintaining the normal physiological functions of cells (Yang et al., 2023; Guo et al., 2024). Increasing evidence shows that crotonylation is closely related to the occurrence and development of tumors. For example, it has been found that there is a competitive antagonistic effect between crotonylation modification and acetylation modification of acetyltransferase KAT7 in colorectal cancer, which can regulate the expression of genes related to procentriole formation, and then affect the growth and proliferation of colorectal cancer cells (Wang M. et al., 2025). In pancreatic cancer, crotonylation modification has been shown to be involved in the regulation of cellular metabolism, affecting the energy metabolism and malignant progression of tumor cells by regulating the activity and stability of metabolic enzymes (Zheng et al., 2023). However, there are still relatively few studies on crotonylation modification in LUAD, and its mechanism of action in the development and prognosis of LUAD is still unclear.

The metabolic reprogramming of tumor cells is an important characteristic that distinguishes them from normal cells, and glycolysis plays a crucial role in the metabolic reprogramming process of lung cancer cells (Lin et al., 2021; Xu et al., 2022). Glycolysis not only provides lung cancer cells with adenosine triphosphate (ATP) required for rapid proliferation, but also produces lactic acid that can acidify the tumor microenvironment and enhance the immune escape ability of tumor cells (Xie et al., 2021; Pu et al., 2024). As an evolutionarily highly conserved multifunctional protein, family with sequence similarity 83 member A (FAM83A) has been proven to promote tumor occurrence and development by regulating different signaling pathways in various tumors such as breast cancer and colorectal cancer (Lee et al., 2012; Liu et al., 2021). However, only a few studies have shown that FAM83A is overexpressed in LUAD tissues, but the specific mechanism of FAM83A in the glycolysis regulatory network of LUAD is not clear. Whether FAM83A affects the metabolism and biological behavior of lung cancer cells by regulating the glycolytic pathway and whether it can serve as a potential target for the treatment of LUAD remain to be further studied.

This study utilized bioinformatics analysis to construct a LUAD prognostic model based on crotonylation genes, and cell experiments were combined to explore the role of hub gene (FAM83A) in regulating glycolysis in lung cancer cells. This study aims to provide new biomarker and potential therapeutic target for prognosis assessment and precision treatment of LUAD, which has important theoretical significance and clinical application value.

## Materials and methods

### Data acquisition

RNA-seq data and clinical information of LUAD patients were downloaded from The Cancer Genome Atlas (TCGA) database (https://portal.gdc.cancer.gov/). The GSE229705 and GSE26939 datasets were downloaded from the Gene Expression Ensemble Database (GEO) (https://www.ncbi.nlm.nih.gov/geo/). The crotonylation-related genes (CRGs) were collected from previous literature (Supplementary Table S1) (Yang et al., 2024).

### Clinical sample collection

The LUAD tissues used in this study and their paired adjacent normal lung tissue samples were collected in accordance with the protocol approved by the Ethics Committee of Mudanjiang Medical University (2024-MYWZ19) after the patients signed the informed consent form. The implementation of this study strictly adhered to the ethical guidelines of the Declaration of Helsinki. The paired adjacent normal lung tissue samples were lung tissues that were more than 2 cm away from the edge of the primary tumor and had no tumor cell infiltration confirmed by intraoperative frozen sections and postoperative paraffin sections pathological examination. Tumor samples cover different pathological grades to ensure that the samples have a certain degree of clinical representativeness.

### Analysis of differentially expressed genes (DEGs)

We used the “limma” R package (Ritchie et al., 2015) to calculate the DEGs between normal and tumor samples. The original sequencing data were converted into a standardized expression matrix after quality control. An experimental design matrix containing grouping information was constructed and a linear model was fitted accordingly. Then, the empirical Bayesian method is used to shrink the variance of genes to stabilize the variance estimate. Finally, the fold change (FC) and p-values were calculated, and an appropriate threshold (|log2(FC)| >2, p < 0.05) was set to screen DEGs. Volcano and heatmap were visualized using the “ggplot2” and “pheatmap” R packages.

### Weighted gene co-expression network analysis (WGCNA)

The “WGCNA” R package was used to construct the gene co-expression network (Langfelder and Horvath, 2008). First, the transcriptome data were obtained and standardized. The correlation between genes was calculated to construct a scale-free network and determine the appropriate soft threshold. Then, hierarchical clustering is carried out based on the similarity of gene expression to divide the co-expression modules and calculate the characteristic values of the modules. Finally, the modules are correlated with the phenotypic data to identify the modules that are significantly related to the phenotype.

### Construction of risk score

The intersection of DEGs and the key module genes of WGCNA was taken to screen the core genes. Survival analysis was performed using the “survminer” R package. The “glmnet” R package (Friedman et al., 2010) was used to fit the Lasso-Cox regression model, and the plot function was used to visualize the position of the optimal lambda curve obtained by the cv.glmnet model. The risk score (riskScore) is calculated by multiplying the expression values of significant genes by their weights and adding them together.

### Functional enrichment analysis

The DEGs list was imported into the DAVID online tool (https://davidbioinformatics.nih.gov/), and the human species database was selected as the background. Subsequently, Gene Ontology (GO) enrichment analysis and Kyoto Encyclopedia of Genes and Genomes (KEGG) pathway enrichment analysis were performed. Finally, the enrichment results are presented using the bubble chart.

### Gene mutation analysis

LUAD patients were divided into high and low subgroups based on the median riskSCore. The mutation data of LUAD patients were downloaded from the TCGA database, and the “maftools” R package (Mayakonda et al., 2018) was used to analyze the gene mutation spectrum and draw the waterfall diagram.

### Gene set variation analysis (GSVA)

The CRGs in tumor samples and normal samples in TCGA-LUAD dataset were analyzed. The analysis was run using the “GSVA” R package to map the gene expression data of TCGA-LUAD tumor samples onto the gene set of CRGs, and the enrichment scores in the gene set were calculated for each sample (Hänzelmann et al., 2013).

### Immune cell infiltration analysis

CIBERSORT (cell-type identification by estimating relative subsets of RNA transcripts) is a calculation method used to evaluate the relative abundance of different cell types in complex mixed tissue samples (Newman et al., 2015). First, prepare the standardized gene expression data and the feature matrix file required by CIBERSORT. Then, the “CIBERSORT” R package is used to calculate the immune cell fraction of each sample. Finally, the changes of immune cells in the high and low riskScore subgroups were compared through difference analysis. TIDE (tumor immune dysfunction and exclusion) is used to assess the possibility of tumor immune escape in the gene expression profile of tumor samples (Jiang et al., 2018). We imported the standardized gene expression data into the TIDE database (http://tide.dfci.harvard.edu/login/) to calculate the TIDE score for each tumor sample.

### Cell lines and cultures

The BEAS-2B, A549 and H1299 cell lines are from the Rosetta Stone Biotech Co., Ltd. (Taiyuan, China). These cells were cultured in Dulbecco Modified Eagle medium (DMEM) containing 10% fetal bovine serum (FBS) and 1% double antibiotics (penicillin-streptomycin) at a temperature of 37 °C with 5% carbon dioxide.

### Knockdown of FAM83A gene

For knockdown of FAM83A, lentivirus-mediated short hairpin RNA (shRNA) was constructed at GenePharma (Suzhou, China). The shRNA sequences targeting human FAM83A gene used in this study were as follows: 5′-GAG​TGT​GGA​AGG​AGA​GAT​ATA-3’ (shFAM83A1) and 5′- AGG​AAA​TTC​GCT​GGC​CAA​ATC-3’ (shFAM83A2). Lung cancer cells were infected using lentivirus-mediated packaging systems. Stable cells with successful FAM83A knockdown were screened using puromycin hydrochloride (Beyotime, Shanghai, China).

### Real-time quantitative PCR (RT-qPCR)

The total RNA of the cell samples was extracted and its concentration and purity were determined (Yin et al., 2024). Prepare the reaction system according to the instructions of the PrimeScript™ RT reagent Kit (Takara, Kusatsu, Japan) to complete reverse transcription and obtain cDNA. Primers were designed based on the target gene and internal reference gene sequences (Supplementary Table S2), and the qPCR reaction system was prepared using SYBR Green qPCR Master Mix (MedChemExpress, Monmouth Junction, United States). The relative expression level of the target gene was analyzed by the 2^−ΔΔCT^ method.

### Cell viability analysis

Stable cell lines A549 and H1299 cells (shCtrl or shFAM83A) were cultured in 96-well plates for 24 h at a density of 5 × 10^3^ cells per well. According to the manufacturer’s protocol, cell viability was detected by 3- (4, 5-dimethylthiazol-2-yl) −2, 5-diphenyltetrazolium bromide (MTT) assay.

### Western blot

Total protein was extracted from cell and tissue samples and the concentration was determined (Hou et al., 2023). Histones were extracted and purified from cells using the histone extraction kit according to the manufacturer’s instructions (Proteintech, Wuhan, China). Proteins were separated by electrophoresis, transferred to polyvinylidene fluoride (PVDF) membranes, and blocked with 5% skim milk powder solution for 1 h at room temperature. Subsequently, primary antibodies pyruvate kinase M2 (PKM2), lactate dehydrogenase A (LDHA), β-actin, Histone H3 (Proteintech, Wuhan, China), H3K27cr (Invitrogen, California, United States), Pan-Crotonyl-K (ABclonal, Wuhan, China) were added and incubated overnight at 4 °C. HRP-labeled secondary antibodies were incubated at room temperature for 1 h. Enhanced chemiluminescence reagent was added and exposed in the gel imaging system, and the gray values of the bands were analyzed by software such as ImageJ.

### Glucose consumption and lactate production

The cell culture supernatant was collected (Yuan et al., 2022). According to the instructions of the glucose detection kit and lactate detection kit (Nanjing Jiancheng Bioengineering Institute), the supernatant was added to the detection well, and the reaction solution of the kit was added in turn. The OD value of each well was determined using an enzyme-linked immunosorbent assay reader at 505 nm (glucose) and 530 nm (lactate). The glucose and lactate concentration in the supernatant was calculated based on the standard curve drawn from the standard substances. The consumption of glucose by the cells was calculated by the difference in glucose concentration before and after culture.

### Chromatin immunoprecipitation (ChIP) assay

ChIP detection was carried out using the ChIP Assay Kit (Beyotime, Shanghai, China) in accordance with the manufacturer’s protocol. The cells were immediately cross-linked in 1% formaldehyde for 15 min, then stopped with glycine and homogenized in cell lysis buffer. Then, the sample was sheared to 100–1,000 bp by ultrasonic treatment. Incubate the cut chromatin with H3K27cr or normal IgG antibodies. Then the precipitated chromosomes are pulled down, purified, and quantified by RT-qPCR. The primers used for ChIP-qPCR detection are listed in Supplementary Table S2.

### Statistical analysis

The analysis was performed using GraphPad Prism (version 8.0) and R (version 4.3.2), and data were expressed as mean ± SD. The differences in variables between two groups were tested by the student t-test, and the differences in variables between more than two groups were tested by one-way analysis of variance and multiple comparison tests. P value <0.05 was considered statistically significant.

## Results

### Identification of key module CRGs by WGCNA

To analyze the role of crotonylation in LUAD patients, based on the GSVA algorithm and through functional classification (Supplementary Table S1 for “Writers”, “Erasers” and “Readers”) analysis of CRGs, we observed that the expression of “Writers” type genes did not change significantly in LUAD tumor tissues compared with adjacent normal tissues. However, the “Erasers” type genes and the “Readers” type genes showed consistent and significant upregulation (Supplementary Figure S1A). This finding indicates that the dysregulation of crotonylation modifications in LUAD is mainly not driven by defects in the “write” phase, but rather by abnormal enhancements in the “erase” and “read” phases, suggesting that crotonylation can regulate LUAD progression.

**FIGURE 1:**
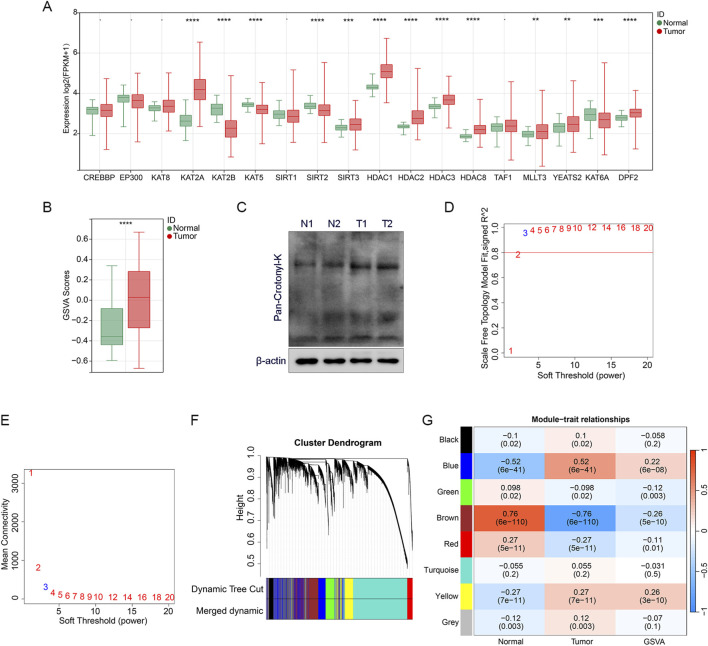
Identification of CRGs genes **(A)** The boxplot shows the differential expression analysis results of CRGs between the tumor and normal samples. **(B)** Differentially expressed crotonylation genes were scored for GSVA enrichment, and the boxplot shows the comparison of GSVA scores between tumor and normal samples. **(C)** Total crotonylation analysis between tumor and normal samples was performed by Western blot. N represents normal samples and T represents tumor samples. **(D,E)** Analysis of the scale-free topology fit index for various soft-thresholding powers to determine the optimal power value for constructing a scale-free co-expression network. **(F)** Cluster dendrogram of genes identified by WGCNA, with different colors representing distinct co-expression modules. **(G)** Heatmap illustrating the correlation coefficients between the identified gene modules and specific clinical traits or crotonylation GSVA scores. Statistical significance is denoted as **p < 0.01, ***p < 0.001, and ****p < 0.0001.

To further explore the role of CRGs in LUAD, we analyzed the differences of 18 CRGs between the tumor group and the control group. A total of 13 CRGs with significant differences were selected for the subsequent analysis (Figure 1A). We calculated the CRGs score using the GSVA algorithm based on the above 13 CRGs, and the CRGs score showed significant difference between the tumor group and the control group, indicating its association with LUAD (Figure 1B). In addition, total crotonylation levels were significantly increased in LUAD tumor tissues compared to adjacent normal tissues as analyzed by Western blot (Figure 1C; Supplementary Figure S1B).

We used CRGs scores as the characteristic data and performed WGCNA analysis to explore the association between gene modules and LUAD traits. Firstly, the soft threshold was systematically screened and β = 3 was determined as the parameter for constructing the scale-free topological network (Figures 1D,E), which ensures that the network conforms to the scale-free property and lays the foundation for subsequent analysis. We constructed a hierarchical clustering tree based on the selected soft threshold β = 3, clustering genes with high co-expression characteristics into the same module and encoding them with different colors (Figure 1F). Subsequently, we plotted the module-trait relationship heatmap of the eight transcriptional modules based on Spearman correlation analysis (Figure 1G). The brown module showed a significant high correlation with LUAD traits, as well as with CRGs scores. Therefore, the brown module was defined as the hub module, and 4377 genes in this module were identified as the hub genes, which may play a key regulatory role in the occurrence and development of LUAD patients.

### Identification of hub DEGs related to crotonylation

**FIGURE 2:**
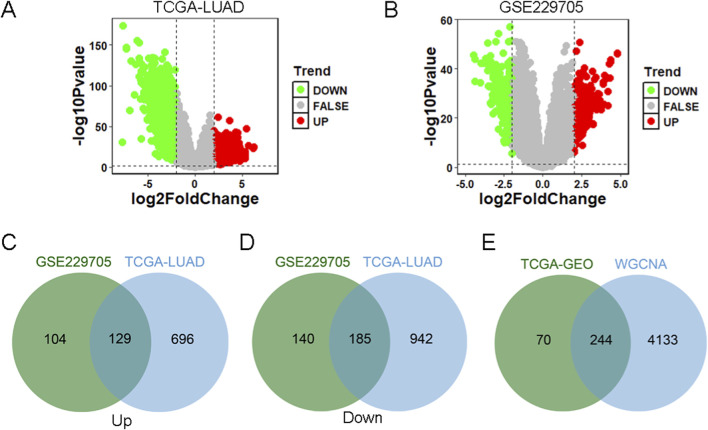
Identification of core differential CRGs **(A)** Volcano plot of DEGs between tumor and normal samples in the TCGA-LUAD dataset. **(B)** Volcano plot of DEGs between tumors and normal samples from the GSE229705 dataset. **(C)** Venn diagram identifying the overlap of upregulated DEGs common to both the TCGA-LUAD and GSE229705 datasets. **(D)** Venn diagram identifying the overlap of downregulated DEGs common to both datasets. **(E)** Venn diagram showing the intersection of consensus DEGs (from TCGA and GEO datasets) with the core module genes identified by WGCNA, leading to the final set of hub CRGs.

We analyzed DEGs between tumor samples and control samples using TCGA database and GEO database, respectively. In the TCGA-LUAD data, there were a total of 1952 DEGs, including 825 upregulated genes and 1,127 downregulated genes (Figure 2A; Supplementary Table S3). In the GSE229705 data, there were a total of 558 DEGs, including 233 upregulated genes and 325 downregulated genes (Figure 2B; Supplementary Table S4). We further took the intersection of the upregulated and downregulated genes from the TCGA-LUAD data and the GSE229705 data, respectively, and obtained a total of 129 intersection upregulated genes and 185 intersection downregulated genes (Figures 2C,D). Subsequently, to identify the central genes associated with crotonylation, we crossed the common DEGs (a total of 314 genes) with the genes of the brown module, which had previously been identified as related to crotonylation through WGCNA. This integration resulted in 244 hub DEGs (Figure 2E), which are hypothesized to play a key role in LUAD through mechanisms involving protein crotonylation. This step-by-step approach not only enhances the reliability of the identified gene set but also provides a molecular basis for understanding the potential involvement of crotonylation in the occurrence and progression of LUAD.

### Construction of prognostic model based on CRGs

**FIGURE 3:**
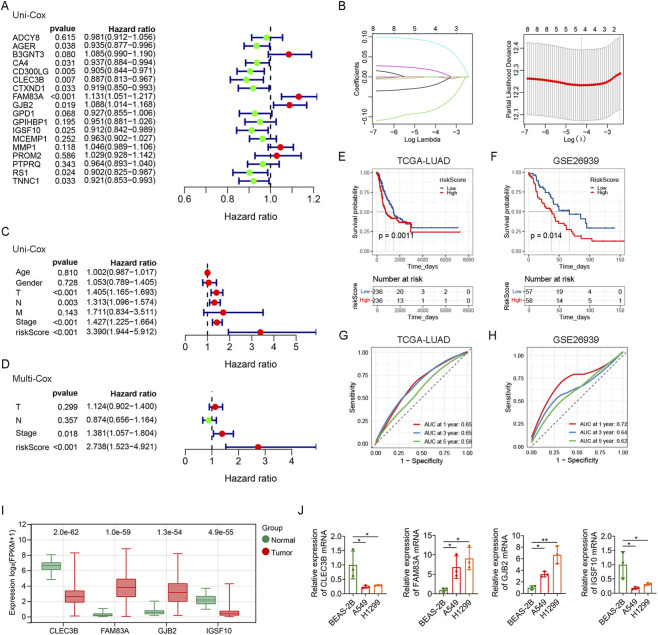
Construction of prognostic model **(A)** Forest plot from univariate Cox analysis of the 18 hub CRGs. **(B)** Profile of Lasso regression coefficients for the variables, showing variable selection. **(C)** Univariate Cox analysis evaluating the prognostic value of clinicopathological factors and riskScore. **(D)** Multivariate Cox analysis of T, N, stage and riskScore. Survival analysis of riskScore in TCGA-LUAD data **(E)** and GSE26939 dataset **(F)**. AUC of riskScore for predicting 1 -, 3 -, and 5-year survival in TCGA-LUAD data **(G)** and GSE26939 dataset **(H)**. **(I)** The expression levels of CLEC3B, FAM83A, GJB2 and IGSF10 in TCGA-LUAD data. **(J)** The mRNA expression levels of CLEC3B, FAM83A, GJB2 and IGSF10 in cell lines (BEAS-2B, A549 and H1299) were determined using RT-qPCR analysis. *p < 0.05, **p < 0.01.

To further construct the prognostic model of genes related to crotonylation, we performed prognostic analysis of 244 hub DEGs related to crotonylation, the expression of 18 genes was closely associated with poor prognosis in LUAD patients by Kaplan-Meier analysis (Supplementary Figure S2). We screened 10 genes (AGER, CA4, CD300LG, CLEC3B, CTXND1, FAM83A, GJB2, IGSF10, RS1 and TNNC1) from 18 genes associated with prognosis by univariate Cox analysis (Figure 3A). We further identified 4 prognostic genes through Lasso regression analysis (Figure 3B). Then, the crotonylation-related risk score of LUAD patients was calculated according to the prognostic characteristics of CLEC3B, FAM83A, GJB2 and IGSF10 genes. The calculation formula is: riskScore = (−0.085 × expression of CLEC3B) + (0.087 × expression of FAM83A) + (0.017 × expression of GJB2) + (−0.023 × expression of IGSF10). LUAD patients were divided into the high riskScore subgroup and the low riskScore subgroup according to the median riskScore. Univariate Cox regression analysis showed that T, N, stage and riskScore were significantly correlated with the prognosis of LUAD patients (Figure 3C). Multivariate Cox regression analysis further confirmed that riskScore was an independent prognostic factor (Figure 3D). Furthermore, the prognosis of LUAD patients with low riskScore was significantly better than that of LUAD patients with high riskScore (Figure 3E). The effect of riskScore was verified using the GSE26939 dataset, and the results were consistent with the TCGA-LUAD data (Figure 3F). In addition, receiver operating characteristic (ROC) curves confirmed that riskScore had diagnostic value for both TCGA-LUAD and GSE26939 datasets (Figures 3G,H). Compared with normal tissues, we found that the expression levels of FAM83A and GJB2 were increased while the expression levels of CLEC3B and IGSF10 were decreased in tumor tissues (Figure 3I). Similar conclusions were obtained in cell experiments. RT-qPCR analysis showed that the expression levels of FAM83A and GJB2 were increased, while the expression levels of CLEC3B and IGSF10 were decreased in lung cancer cells (A549 and H1299) compared with lung epithelial cells (BEAS-2B) (Figure 3J). Together, we successfully constructed and validated a robust prognostic model based on four CRGs. This model can not only effectively predict the survival outcome of LUAD patients, but its risk score is also an independent prognostic factor.

### Functional enrichment and gene mutation landscape analysis of riskScore subgroups

**FIGURE 4:**
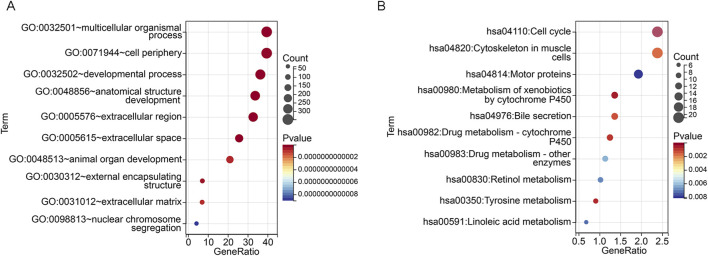
Functional enrichment analysis based on riskScore subgroups **(A)** GO enrichment analysis bubble chart showing significant enrichment of biological processes, cellular components and molecular functions between high and low riskScore groups. **(B)** Bubble plot of KEGG pathway enrichment analysis, highlighting differential activation of signaling and metabolic pathways between riskScore subgroups.

To further analyze the molecular mechanisms regulating LUAD progression in the high and low riskScore subgroups, we performed functional enrichment analysis using DEGs from the high and low riskScore subgroups (Supplementary Table S5). The top 10 pathways of GO and KEGG pathways are shown in Figures 4A,B. GO analysis revealed that the pathways were mainly concentrated in multicellular organismal process, cell periphery and developmental process, etc. It is suggested that the differences among these subgroups may involve key biological events such as intercellular communication, maintenance of cell structure and tissue development. KEGG pathway analysis indicated that the differentially expressed genes were significantly enriched in pathways such as cell cycle, drug metabolism, and retinol metabolism, suggesting that between the high-riskScore and low-riskScore groups, there may be significant differences in cell proliferation regulation, drug metabolism capacity, and vitamin A metabolism, etc. These findings provide important clues for further understanding the pathogenesis and potential therapeutic targets of LUAD. Functional enrichment analysis revealed the differences among various risk groups in key biological processes such as cell proliferation and metabolism, which might be the intrinsic reason for their different prognoses.

**FIGURE 5:**
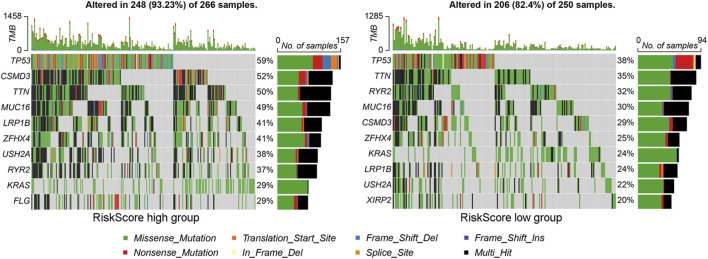
Gene mutation landscape analysis of riskScore subgroups. The oncopprint plot shows the mutation profiles (including missense mutations, indels and other variant types) of the top 10 significantly mutated genes in the (left) high-riskScore subgroup and (right) low-riskScore subgroup. Tumor mutation burden and specific mutation patterns were visualized for comparison.

We further explored the gene mutations in the high and low riskScore subgroups and found that the number of mutation samples in the high riskScore subgroup was higher than that in the low riskScore subgroup (93.23% vs. 82.4%). The top 10 genes with the highest mutation rates in different riskScore subgroups are shown in Figure 5, among which the mutation rates of TP53, TTN and RYR2 were higher than 30% in both groups of patients, and the most significant difference was in TP53 mutation (high riskScore subgroup (59%) vs. low riskScore subgroup (38%)). These results indicate that the high-riskScore group has a higher tumor mutation burden and more frequent TP53 mutations.

### Immune cell analysis of riskScore subgroups

**FIGURE 6:**
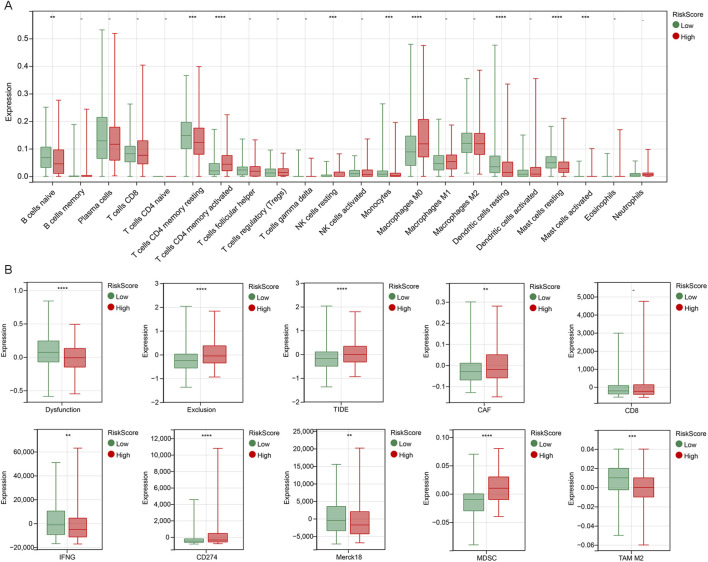
Immune microenvironment analysis of riskScore subgroups **(A)** A box plot comparing the infiltration levels of 22 types of immune cells between the high-riskScore group and the low-riskScore group, estimated by the CIBERSORT algorithm. **(B)** TIDE analysis of riskScore subgroup. **p < 0.01, ***p < 0.001, ****p < 0.0001.

The occurrence and development of tumors are closely related to the immune system, among which tumor immune cells play a key role in the immune surveillance, immune escape and immunotherapy of tumors. We calculated 22 immune cells using the CIBERSORT algorithm, and we found that the high riskScore subgroup had increased expression of activated memory CD4 T cells, resting NK cells, M0 macrophages and activated mast cells, and less expression of naive B cells, resting memory CD4 T cells, monocytes, resting dendritic cells and resting mast cells compared to the low riskScore subgroup (Figure 6A).

TIDE is an algorithm used to predict the response of cancer patients to immune checkpoint inhibitors. We used the TIDE algorithm to evaluate the possibility of tumor immune escape in the gene expression profiles of tumor samples in the high and low riskScore subgroups. Compared with the low riskScore subgroup, the TIDE score, exclusion score, cancer-associated fibroblast (CAF), CD274 and myeloid-derived suppressor cell (MDSC) in the high riskScore subgroup were significantly higher, while the IFNG was lower, indicating that the immune escape potential of patients in the high riskScore group was increased, and the immune checkpoint suppression therapy efficacy may be poor. In addition, it is possible that too small sample size resulted in higher dysfunction score, Merck18 and tumor-associated macrophages (TAM) M2 for patients in the low riskScore subgroup (Figure 6B). Therefore, the tumor immune microenvironment in the high-riskScore subgroup shows a stronger immunosuppressive state. These findings suggest that the risk prognostic model can reflect the immune characteristics of LUAD and may have predictive value for the efficacy of immunotherapy.

### FAM83A promotes aerobic glycolysis of tumor cells

**FIGURE 7:**
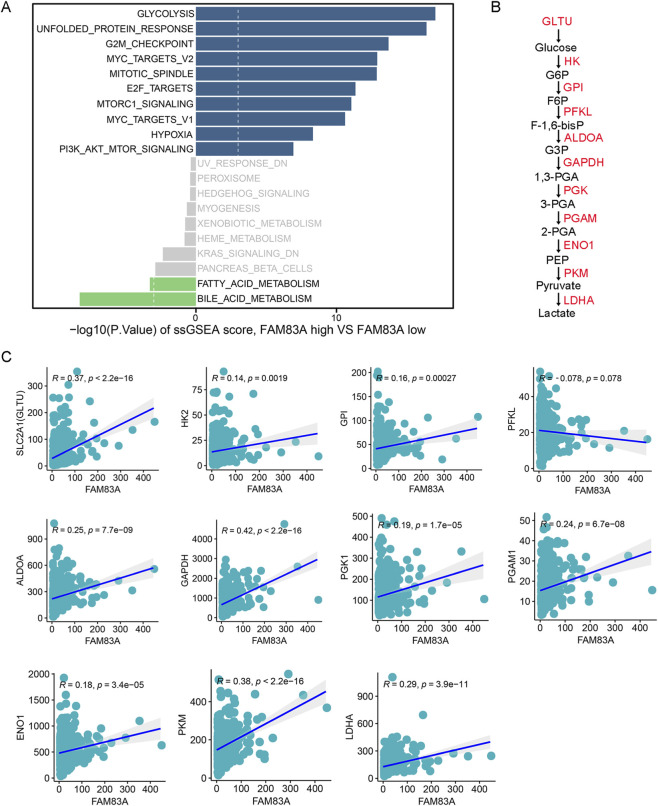
FAM83A promotes glycolysis by bioinformatics analysis **(A)** Bar plots of the top 10 hallmark functional pathways between FAM83A-high and FAN83A-low groups in TCGA-LUAD patients were determined by ssGSEA. **(B)** Flowchart of key enzymes and products in glycolysis. **(C)** Correlation analysis between FAM83A and key glycolytic enzymes.

To further analyze the molecular mechanisms of the candidate genes regulating LUAD progression, the FAM83A gene was selected for subsequent analysis as the differences were most obvious (Figure 3I). We used ssGSEA algorithm to perform functional enrichment scores for LUAD patients, and divided patients into high and low expression groups based on the median of FAM83A gene. The analysis results indicated that high expression of FAM83A promoted glycolysis (Figure 7A). The main enzymes and intermediate metabolites involved in the glycolysis process are shown in Figure 7B. We observed FAM83A weak but statistically significant positive correlation between SLC2A1 (also known as GLTU), HK2, GPI, ALDOA, GAPDH, PGK1, PGAM1, ENO1, PKM and LDHA, suggesting that there might be some kind of trend connection between the two, but this linear association is not strong (Figure 7C). Together, FAM83A promotes tumor progression by regulating tumor cell glycolysis.

**FIGURE 8:**
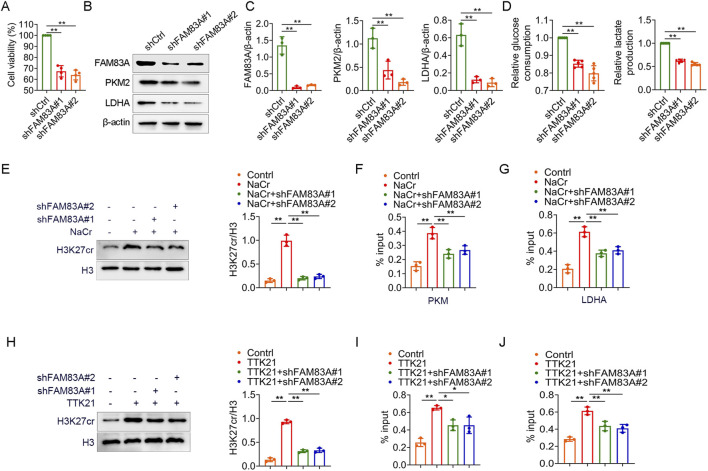
FAM83A promotes glycolysis by A549 cell experiments **(A–D)** A549 cells were transfected with control shRNA (shCtrl) and FAM83A shRNA (shFAM83A). **(A)** MTT analysis of cell proliferation. **(B,C)** The expression levels of FAM83A, PKM2 and LDHA were detected by Western blot, and the protein density was quantified by densitometry. **(D)** Glucose consumption and lactate production. **(E–G)** A549 cells were transfected with shFAM83A and treated with 10 mM NaCr for 24 h. **(E)** The expression levels of H3K27cr and H3 were detected by Western blot, and the protein density was quantified by densitometry. **(F,G)** ChIP-qPCR experiments were performed using H3K27cr antibody to detect the transcript levels of PKM and LDHA. **(H–J)** A549 cells were transfected with shFAM83A and treated with 1 mM TTK21 for 24 h. **(H)** The expression levels of H3K27cr and H3 were detected by Western blot, and the protein density was quantified by densitometry. **(I,J)** ChIP-qPCR experiments were performed using H3K27cr antibody to detect the transcript levels of PKM and LDHA. *p < 0.05, **p < 0.01.

**FIGURE 9:**
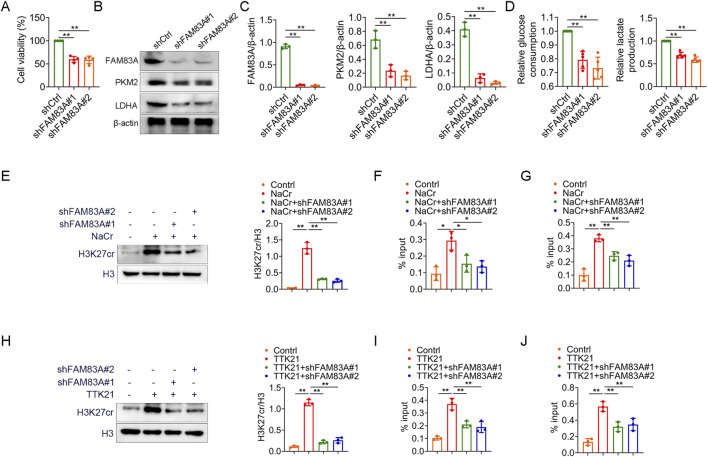
FAM83A promotes glycolysis by H1299 cell experiments **(A–D)** H1299 cells were transfected with control shRNA (shCtrl) and FAM83A shRNA (shFAM83A). **(A)** MTT analysis of cell proliferation. **(B,C)** The expression levels of FAM83A, PKM2 and LDHA were detected by Western blot, and the protein density was quantified by densitometry. **(D)** Glucose consumption and lactate production. **(E–G)** H1299 cells were transfected with shFAM83A and treated with 10 mM NaCr for 24 h. **(E)** The expression levels of H3K27cr and H3 were detected by Western blot, and the protein density was quantified by densitometry. **(F,G)** ChIP-qPCR experiments were performed using H3K27cr antibody to detect the transcript levels of PKM and LDHA. **(H–J)** H1299 cells were transfected with shFAM83A and treated with 1 mM TTK21 for 24 h. **(H)** The expression levels of H3K27cr and H3 were detected by Western blot, and the protein density was quantified by densitometry. **(I,J)** ChIP-qPCR experiments were performed using H3K27cr antibody to detect the transcript levels of PKM and LDHA. *p < 0.05, **p < 0.01.

**FIGURE 10:**
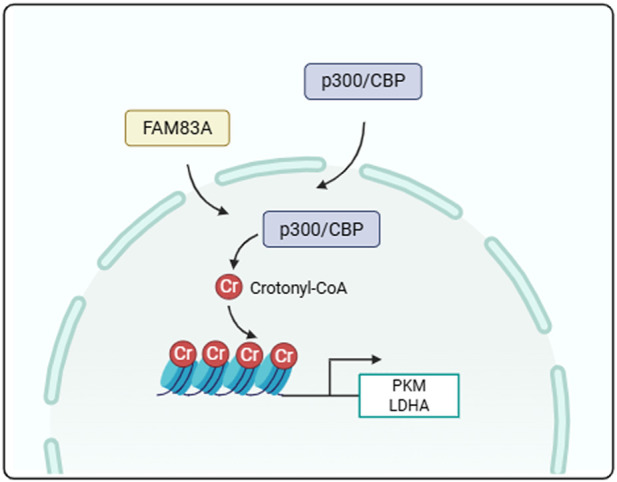
Schematic diagram of mechanism. FAM83A promotes tumor cell glycolysis by regulating the crotonylation of histones.

We further confirmed the role of FAM83A in lung cancer cells by cell experiments. FAM83A was knocked down in A549 cells by shRNA (Supplementary Figure S3A), and the knockdown of FAM83A significantly inhibited cell proliferation (Figure 8A) and reduced global protein crotonylation levels (Supplementary Figure S3B), suggesting a broad impact on this post-translational modification. Meanwhile, we also treated BEAS-2B, A549 and H1299 cells with NaCr (a deacetylase inhibitor), and compared with normal BEAS-2B cells, NaCr treatment increased FAM83A expression in tumor cells as determined by Western blot (Supplementary Figure S3C). Knockdown of FAM83A also significantly inhibited the expression of key glycolytic enzymes PKM2 and LDHA by Western blot analysis (Figures 8B,C). In addition, increased glucose consumption and lactate production are important markers of glycolysis, which were significantly inhibited by FAM83A knockdown (Figure 8D). We next explored the mechanism linking FAM83A to histone crotonylation. The treatment with NaCr effectively increased the H3K27cr level. Notably, this enhancement was entirely dependent on FAM83A, as knockdown of FAM83A abolished the NaCr-induced H3K27cr increase (Figure 8E). ChIP-qPCR analysis using H3K27cr antibody revealed that the NaCr-induced transcriptional activation of PKM and LDHA was critically dependent on FAM83A (Figures 8F,G). The histone acetyltransferase p300 also functions as a robust histone crotonyltransferase. It catalyzes histone crotonylation in a crotonyl-CoA-dependent manner, and this activity is integral to the regulation of gene transcription (Gao et al., 2024). TTK21 is an agonist of P300, we treated A549 cells with TTK21 and/or shRNA FAM83A, and knockdown of FAM83A significantly inhibited TTK21-induced H3K27cr expression (Figure 8H). ChIP-qPCR analysis further showed that FAM83A was required for TTK21-driven transcriptional upregulation of PKM and LDHA (Figures 8I,J). These results were consistently validated in H1299 cells (Figure 9; Supplementary Figures S3A,B). Collectively, our findings establish FAM83A as a master regulator that integrates histone crotonylation signaling to drive transcriptional reprogramming of glycolytic metabolism in lung cancer cells (Figure 10).

## Discussion

In recent years, the role of metabolic reprogramming in the occurrence and development of tumors has attracted increasing attention. As one of the key mechanisms regulating cellular metabolism, protein post-translational modification (such as crotonylation) has gradually become a research hotspot (Zheng et al., 2023; Wang et al., 2021). It is worth noting that although both this study and the research of Bowen Hu et al. focused on the role of crotonylation in LUAD, there were significant differences in the analytical concepts. Bowen Hu et al. identified candidate genes by integrating DEGs in tumor and normal samples, as well as the intersection of WGCNA core module genes and CRGs (Hu et al., 2025). In this study, GSVA was used to analyze and quantify the crotonylation level of each sample, and it was used as the trait parameter for screening core module genes in WGCNA. This methodological difference leads to different conclusions from the two studies and enriches our understanding of the regulatory network of crotonylation from different perspectives.

Based on the above analysis strategies, this study constructed a prognostic risk assessment model for LUAD patients. The results showed that the prognosis of patients in the high riskScore group was significantly worse, suggesting that crotonylation may promote the malignant progression of tumors by affecting metabolic pathways. This finding echoes the research of Hou et al. in colorectal cancer, which confirmed that crotonylation can drive tumorigenesis by regulating the activity of key glycolytic enzymes (Hou et al., 2024). This study further expands the role of crotonylation in LUAD and provides new clues for exploring the molecular mechanism of crotonylation.

At the tumor microenvironment level, the high riskScore group showed significant immunosuppressive characteristics, suggesting that crotonylation-related genes may affect the immune response by regulating the function of immune cells. In recent years, multiple studies have emphasized that lactic acid accumulation and acidic microenvironment caused by abnormal tumor metabolism can significantly inhibit T cell activity (Kumagai et al., 2022; Apostolova and Pearce, 2022; Gao et al., 2022). This study hypothesized that the enhanced glycolytic activity in the high riskScore group may promote immune escape by acidizing the microenvironment or directly interfering with the metabolic state of immune cells. This view is highly consistent with the conclusion of Brand et al. That LDHA-mediated lactic acid metabolism drives immunosuppression (Brand et al., 2016). Further revealing the role of crotonylation in the expression or functional regulation of immune checkpoints will help to understand the mechanism of immunotherapy resistance and provide a theoretical basis for the combined application of crotonylation inhibitors and immune checkpoint inhibitors.

Functional enrichment analysis further revealed that metabolic pathway-related genes were significantly enriched in the high riskScore group, suggesting that metabolic reprogramming is a key factor leading to poor prognosis in patients. A large number of studies have shown that tumor cells rely on glycolysis to achieve rapid proliferation and bioenergy supply (Paul et al., 2022; Qiao et al., 2024; Wu et al., 2023). This study found that FAM83A, as a key regulatory factor, can promote the progression of LUAD by facilitating glycolysis. Furthermore, our pathway analysis revealed that FAM83A activates glycolysis while inhibiting fatty acid metabolism. This most likely reflects a cellular intrinsic energy metabolic program switch. In the context of the classic Warburg effect, cancer cells tend to rely on glycolysis for rapid energy supply and accordingly reduce the time-consuming fatty acid oxidation. FAM83A might be at the upstream node of this metabolic transition. A reasonable explanation is that the crotonylation modification network regulated by FAM83A simultaneously covers the key enzymes of glycolysis and fatty acid metabolism. This discovery corroborates the reports in the existing literature on the role of FAM83A in tumors, but there are also significant differences. For instance, Chen et al. reported that FAM83A-AS1 enhances glycolysis in LUAD by activating the HIF-1α signaling pathway (Chen et al., 2022), while this study directly confirmed that FAM83A itself plays a core role in the metabolic reprogramming of LUAD glycolysis. In addition, compared with the mechanism by which FAM83A promotes tumor proliferation by regulating Wnt/β-catenin, EGFR, MAPK, EMT, and other signaling pathways and physiological processes in models of pancreatic cancer, lung cancer, breast cancer, and other malignant tumors (Zhao et al., 2024), this study reveals a new function of FAM83A in regulating energy metabolism in LUAD, expanding the understanding of the oncogenic mechanism of this gene. It is worth noting that FAM83A may act as a downstream effector molecule of crotonylation modification, integrating upstream signals and amplifying metabolic remodeling effects. Our mechanistic exploration reveals a new regulatory axis: FAM83A regulates the transcription of key genes in glycolysis by regulating the crotonylation modification at histone H3K27, which ultimately drives metabolic reprogramming. Therefore, targeting FAM83A and its related metabolic pathways not only interferes with tumor energy supply but also may reverse the immunosuppressive state, demonstrating significant translational potential.

Although this study systematically revealed the prognostic value of CRGs and the key role of FAM83A in glycolysis, there are still several limitations. Firstly, our functional verification mainly relies on *in vitro* cell line models. Although we used two representative LUAD cell lines and obtained consistent results, the cell line model could not fully simulate the complex microenvironment and cellular heterogeneity of tumors *in vivo*. Secondly, although our findings based on the TCGA database are statistically significant, further validation is still needed in prospective clinical cohorts or more complex preclinical models (such as patient-derived organoids or xenograft models) to confirm the clinical translational potential of FAM83A as a biomarker or therapeutic target. Thirdly, the specific molecular mechanism by which FAM83A regulates glycolysis has not been fully clarified. Subsequently, CRISPR gene editing technology and metabolomics methods can be combined to reveal its downstream signaling network and effector molecules. In addition, the cross-dialogue between crotonylation and other metabolism-related modifications (such as acetylation and succinylation), as well as their synergistic or antagonistic effects in metabolic reprogramming, are also directions worthy of in-depth exploration in the future. From a broader perspective, this study not only deepens the understanding of the role of crotonylation in the metabolic regulation of lung cancer, but also lays a theoretical foundation for the development of combined treatment strategies targeting metabolic modifications, which has potential clinical translational value.

## Conclusion

In conclusion, this study for the first time linked crotonylation-related genes to the prognosis and metabolic reprogramming of LUAD, and revealed the immunosuppressive characteristics of the high riskScore group, confirming the role of FAM83A in driving tumor progression by promoting glycolysis. These findings not only provide new markers for the prognostic evaluation of LUAD, but also provide potential targets for therapeutic strategies targeting the metabolic-immune axis. Future studies can be combined with preclinical models to explore the application value of inhibitors targeting crotonylation and glycolysis pathways in the treatment of LUAD patients.

## Data Availability

The original contributions presented in the study are included in the article/Supplementary Material. Further inquiries can be directed to the corresponding authors.
