# Supplementary material for: A putative prognostic model for lung adenocarcinoma based on crotonylation-related genes by bioinformatics and experimental verification
Source: Front Cell Dev Biol. 2026 Feb 17;14:1639773. doi: 10.3389/fcell.2026.1639773 (PMC12953391; doi:10.3389/fcell.2026.1639773)
Supplement: Supplementary file 3 [file DataSheet1.doc]

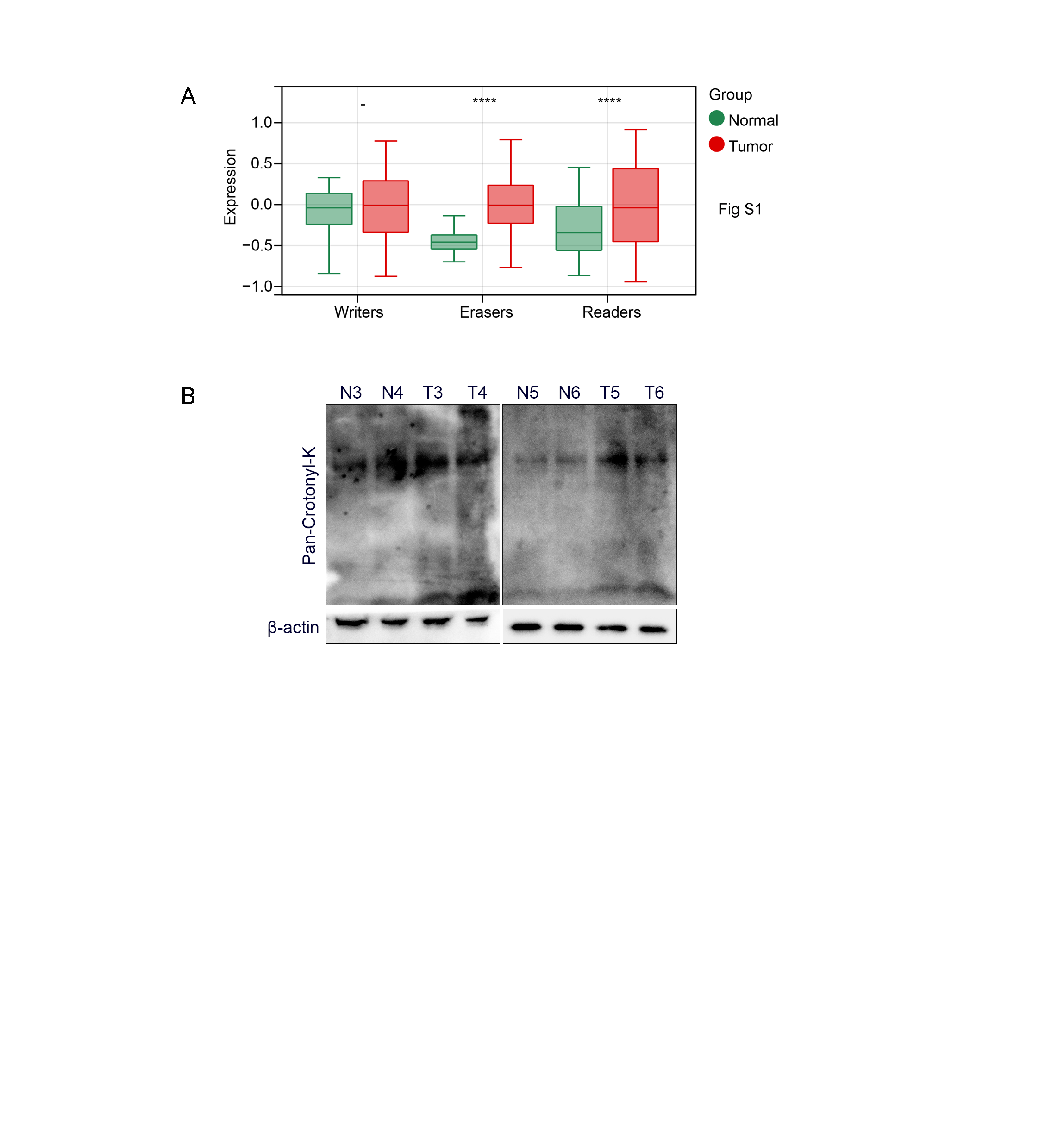


**Figure S1**

**Crotonylation analysis**

A. Based on the GSVA algorithm, the box plot presents the functional classification (" Writers ", "Erasers" and "Readers") analysis of CRGs in normal and tumor samples. **** p<0.0001.

B. Total crotonylation analysis between tumor and normal samples was performed by western blot. N represents normal samples and T represents tumor samples.


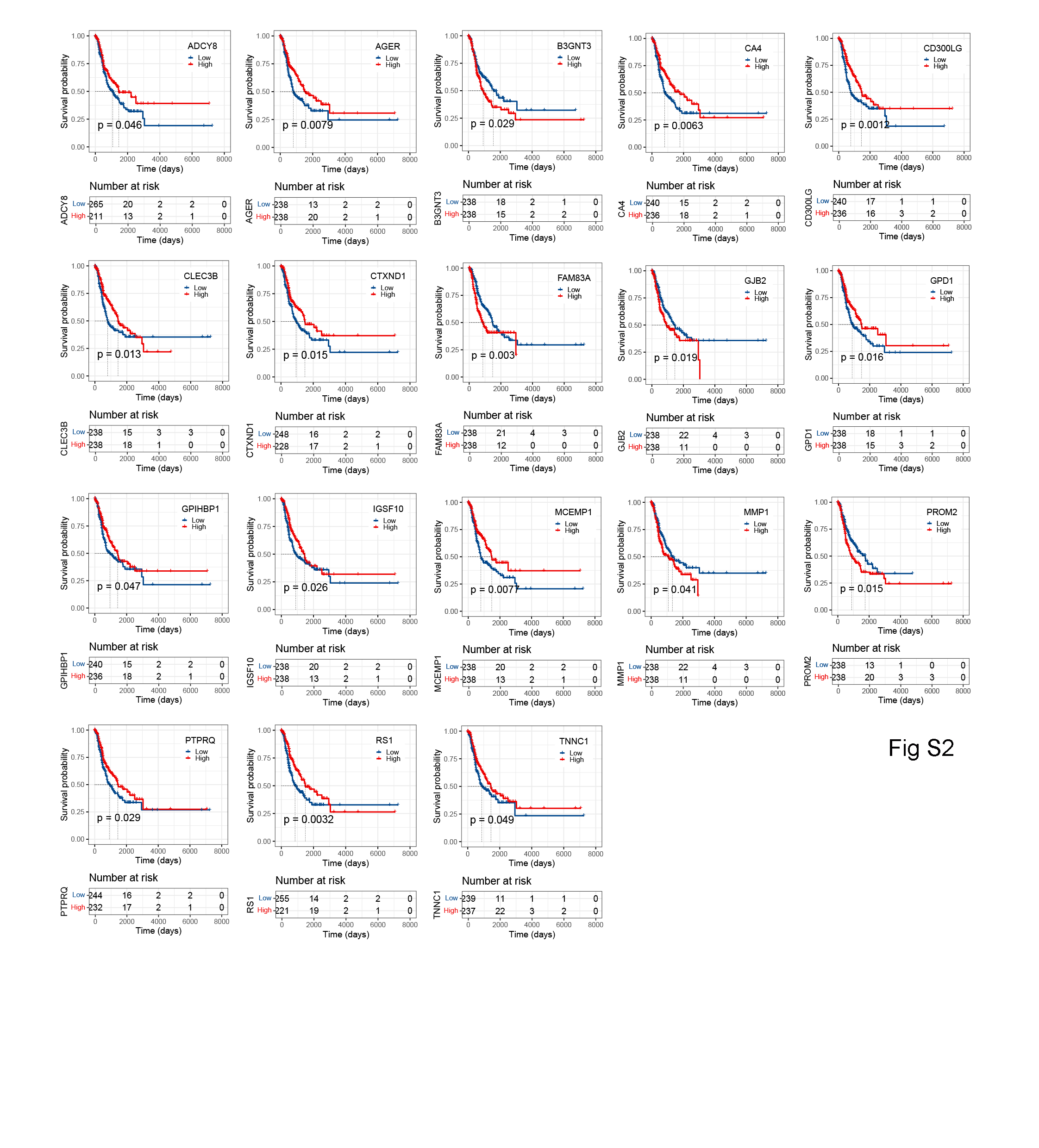


**Figure S2**

**Survival analysis of** **18 CRGs.**

Kaplan-Meier survival analysis of 18 CRGs in TCGA-LUAD data.


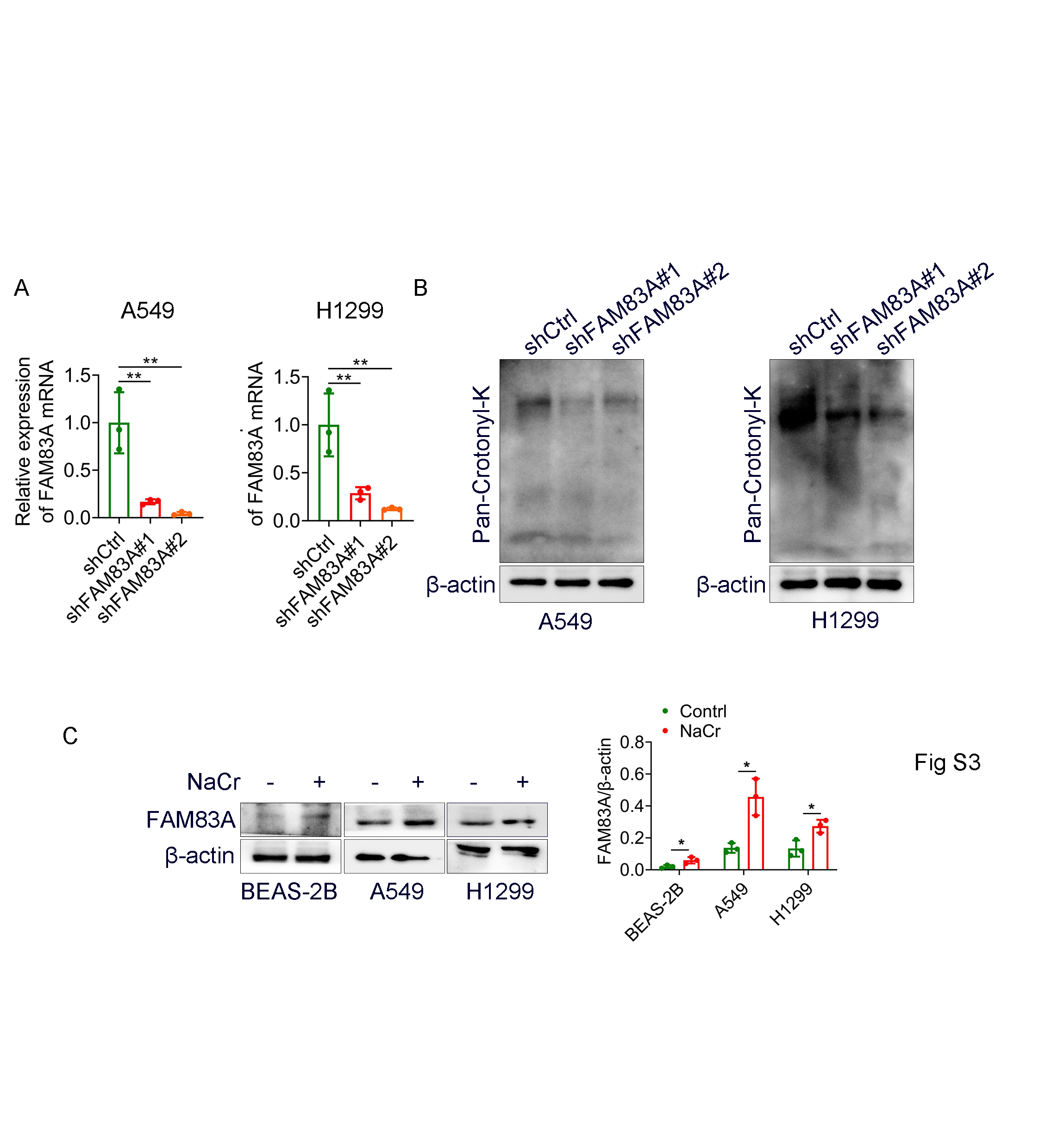


**Figure S3**

**FAM83A can regulate crotonylation.**

(A, B) A549 and H1299 cells were transfected with control shRNA (shCtrl) and FAM83A shRNA (shFAM83A). (A) The mRNA expression levels of FAM83A were determined using RT-qPCR analysis. (B) Total crotonylation analysis was detected by western blot. (C) BEAS-2B, A549 and H1299 cells were treated with 10 mM NaCr for 24 hours. The expression levels of FAM83A were detected by western blot, and the protein density was quantified by densitometry. * p<0.05. ** p<0.01.
